# Possible familial predisposition in superior mesenteric artery syndrome with surgical management: A case report

**DOI:** 10.1097/MD.0000000000042613

**Published:** 2025-05-23

**Authors:** Mohammad Maraqah, Mohammad Farid Al Tamimi, Sara Abu Aram, Shukran Hmedat, Maimana Abueisha, Yazan Hmeedat

**Affiliations:** a College of Medicine, Hebron University, Hebron, West Bank, Palestine; b Department of Surgery, Palestine Red Crescent Society (PRCS) Hospital, Hebron, Palestine; c College of Medicine, Palestine Polytechnic University, Hebron, Palestine.

**Keywords:** duodenal obstruction, familial predisposition, genetic susceptibility, intestinal obstruction, pediatric gastrointestinal disorders, SMA syndrome, superior mesenteric artery syndrome, Wilkie’s syndrome

## Abstract

**Rationale:**

Superior mesenteric artery (SMA) Syndrome is a rare gastrointestinal disorder caused by compression of the third part of the duodenum between the SMA and the aorta due to a reduced aortomesenteric angle. While most cases are sporadic, familial patterns suggest a possible genetic predisposition. This report presents a case of SMA syndrome in a 16-year-old male, with a familial occurrence, raising suspicion of possible genetic correlation.

**Patient concerns:**

A 16-year-old male presented with chronic food intolerance, bilious vomiting, early satiety, and epigastric pain for over 4 years, along with significant weight loss (37–30 kg). His symptoms were exacerbated postprandially and improved with positional changes.

**Diagnoses:**

Ultrasound raised suspicion of SMA compression, which was confirmed by contrast-enhanced computed tomography imaging showing a reduced aortomesenteric angle of 18.2° consistent with SMA syndrome.

**Interventions:**

Conservative management, including nutritional support and proton pump inhibitors, failed to alleviate symptoms. Therefore, the patient underwent laparoscopic Roux-en-Y gastrojejunostomy without complications.

**Outcomes:**

The patient tolerated a full fluid diet postoperatively, experienced regular bowel movements, and was discharged 3 days after surgery. Follow-up care included imaging, endoscopy, and laboratory testing to monitor nutritional and metabolic status.

**Lessons:**

This case highlights the potential familial predisposition to SMA syndrome, emphasizing the need for further studies on genetic components. It also emphasizes the importance of early diagnosis and individualized management strategies, including surgical interventions, for refractory cases.

## 1. Introduction

Superior mesenteric artery (SMA) Syndrome, also known as Wilkie’s syndrome, is a rare but potentially life-threatening gastrointestinal disorder caused by the compression of the third portion of the duodenum between the SMA and the abdominal aorta. This compression occurs when the aortomesenteric angle decreases, a space typically maintained by a cushion of mesenteric fat. In healthy individuals, the angle between the SMA and the aorta ranges from 38° to 68° and aortomesenteric distance is 10 to 28 mm, allowing for unobstructed passage of the duodenum. However, in SMA syndrome, this angle narrows to <28° and distance to 2 to 8 mm, leading to partial or complete duodenal obstruction. This results in symptoms such as postprandial pain, nausea, vomiting, early satiety, and significant weight loss with severity varying depending on the degree of duodenal compression.^[1–3]^

Most cases are sporadic. However, some studies suggest a genetic predisposition to SMA syndrome, particularly in individuals with a congenitally shortened ligament of Treitz, which may predispose them to obstruction by the SMA more than the general population.^[4]^

SMA syndrome is often misdiagnosed due to its nonspecific symptoms, making its detection challenging. Early diagnosis is important in order to prevent serious complications such as severe malnutrition, electrolyte imbalances, peptic ulcers, and, in rare cases, duodenal perforation or necrosis.^[5,6]^

In our case, we present a 16-year-old male patient who presented with chronic intolerance to food, vomiting, and significant weight loss of 7 kg. His condition was diagnosed through supporting findings on computed tomography (CT) scan for SMA syndrome. Our patient required laparoscopic gastrojejunostomy due to failure of initial conservative management, which had included nutritional support and pharmacological therapy with proton pump inhibitors. Notably, the patient’s brother had been diagnosed with the same condition, raising suspicion of a familial predisposition to SMA syndrome. This case highlights the possibility of SMA syndrome having an inheriting pattern, particularly when a familial occurrence is noted.

## 2. Case presentation

A 16-year-old male presented to the surgery clinic complaining of a variety of symptoms, including heartburn, food intolerance, chronic bilious vomiting, early satiety, and postprandial abdominal pain localized to the epigastrium for over 4 years. He had significant weight loss (from 37 to 30 kg) over this period of time. The pain was exacerbated by meals and relieved by lying prone or in a left lateral decubitus position. These symptoms led to noticeable malnutrition, generalized weakness, and visible wasting, with no history of diarrhea, fever, or abdominal trauma.

The patient’s family history was notable for a sibling who had been diagnosed with SMA syndrome. The sibling, a 21-year-old male, had undergone surgery for definitive treatment but unfortunately experienced severe vomiting, episodes of loss of consciousness, and required a tracheostomy. He remained hospitalized until his death.

Considering the patient’s investigations, the initial laboratory tests, including a complete blood count, C-reactive protein, and liver enzymes, were all within normal ranges. An abdominal ultrasound was done and revealed a mildly reduced angle between the SMA and aorta. Following this, an esophagogastroduodenoscopy was performed, revealing Grade B reflux esophagitis with ulceration at the Z-line and a dilated second part of the duodenum. Biopsies obtained during the procedure indicated chronic gastritis, mild chronic nonspecific duodenal inflammation, and no evidence of malignant transformation or *H pylori*.

**Figure 1.**
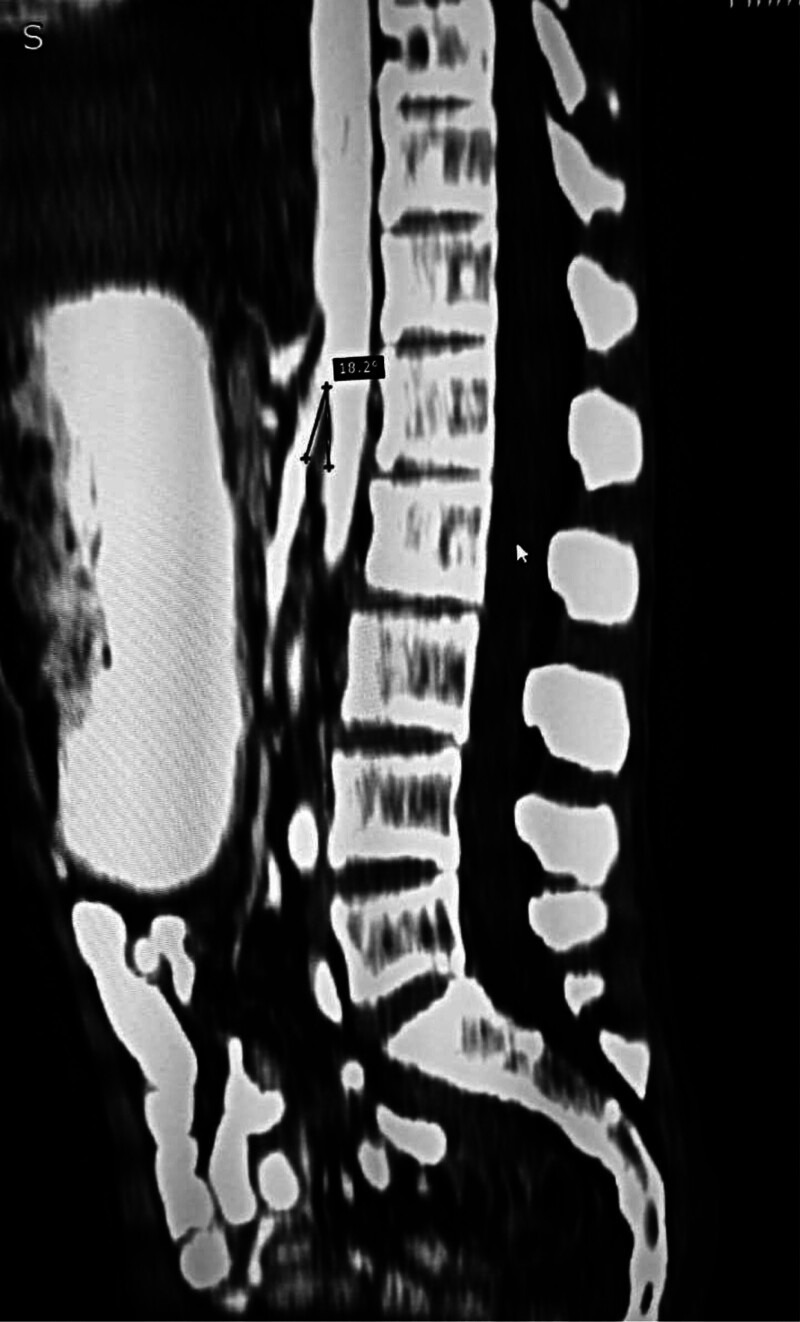
Contrast-enhanced CT scan of the abdomen showing an angle of 18.2° between the superior mesenteric artery (SMA) and the aorta. CT = computed tomography.

Then a contrast-enhanced CT scan was done and subsequently confirmed a narrowed aortomesenteric angle measuring 18.2° and compressed third part of the duodenum with significant gastric distension, confirming the diagnosis of SMA syndrome (Fig. 1).

Conservative management, including nutritional support consisting of frequent, small, high-calorie meals rich in proteins and healthy fats, supported by oral appetite stimulants; postural therapy such as positioning in the prone, knee-chest, or left lateral decubitus positions after meals; and a course of proton pump inhibitors, was initially attempted for 6 weeks, but failed to alleviate the patient’s symptoms. As a result, surgical intervention was recommended. The patient’s BMI was 9.8 kg/m² recorded the day before surgery. The patient subsequently underwent a laparoscopic Roux-en-Y gastrojejunostomy, which was performed without complications.

Postoperatively, he tolerated a full fluid diet, had regular bowel movements, and was discharged on the third postoperative day with recommendations for electrolyte replacement. During the 4-month follow-up period, he demonstrated remarkable clinical improvement. His weight increased from 30 to 38 kg, and his symptoms of vomiting and postprandial discomfort resolved completely.

## 3. Discussion and conclusions

SMA syndrome, while rare, remains a clinically significant cause of intestinal obstruction, particularly in patients with predisposing factors such as rapid weight loss, prolonged immobilization, or anatomical anomalies.^[7]^ It’s overall prevalence in unknown, but some studies had estimated it in the general population to has an approximate range between 0.013% and 0.78% based on radiographic findings, with higher incidences reported in specific groups, such as 2.73% in patients hospitalized for anorexia nervosa and 10.8% in individuals with functional dyspepsia.^[8,9]^

Causes of this syndrome could be either congenital or acquired. The most common cause is loss of mesenteric fat, which serves as a protective cushion and prevents vascular compression. This fat loss is often secondary to rapid weight loss, as seen in conditions of malignancy, burns, chronic infections and malabsorptive states.^[10]^ Congenital anatomical variations, such as a short ligament of Treitz, incomplete rotation of the duodenum, or a low origin of the SMA, can reduce the aortomesenteric angle and distance as well.^[11]^ Given our patient’s family history, including a sibling with the same condition, SMA syndrome may have a genetic predisposition. Although SMA syndrome is not yet classified as an inherited disorder due to insufficient evidence, case reports, including one involving twin and another involving a patient and her mother, suggest a potential familial pattern or genetic link.^[12,13]^

The nonspecific nature of symptoms associated with SMA syndrome often leads to its omission from the differential diagnosis, making it an overlooked condition. In this case, we emphasize the importance of considering SMA syndrome in patients presenting with vague gastrointestinal complaints. Diagnosis relies on clinical symptoms supported by radiographic findings suggestive of duodenal obstruction. X-ray with barium can detect stenosis in the duodenum, while gastroscopy may reveal dilatation, liquid stasis, and antiperistaltic waves. Contrast-enhanced CT is the gold standard for diagnosis, providing detailed anatomical visualization.^[1]^ Therefore, we based our diagnosis on contrast-enhanced CT and esophagogastroduodenoscopy findings, in conjunction with the patient’s clinical presentation.

Although ultrasonography offers a radiation-free assessment of SMA angle and duodenal passage, its use remains limited due to its inability to provide detailed anatomical visualization comparable to CT or barium studies. The advantage of CT over ultrasonography is its ability to demonstrate additional pathologies along with dilatation of the stomach and the duodenum.^[14]^

Considering management strategies for SMA syndrome, they typically begin with conservative measures, which include postural adjustments (e.g., left lateral or knee-to-chest positioning), nasogastric decompression, and nutritional support. The primary objective of conservative management is to increase mesenteric fat deposition, thereby widening the aortomesenteric angle and relieving duodenal compression, these measures are successful in approximately 70% of cases. However, surgical intervention including gastrojejunostomy, gastroduodenostomy and duodenojejunostomy via laparoscopic techniques becomes necessary for refractory cases with success rates that ranges from 80% to 100% in some studies.^[15]^ They are considered the definitive treatment for unresponsive patients, as in our patient who kept having symptoms despite the initial conservative management steps.

In conclusion, our case points out the potential role of genetic susceptibility in the development of SMA syndrome, as demonstrated by the occurrence of the condition in both the patient and his sibling. It also important to notice the possibility of using the ultrasound as a supportive, noninvasive imaging modality in the initial assessment of SMA syndrome, aiding in early detection and guiding further diagnostic evaluation. We think that further studies are necessary to explore genetic predispositions and possible familial patterns of this syndrome.

## Acknowledgments

We would like to express our gratitude to the patient and their family for their participation in this study.

## Author contributions

**Data curation:** Mohammad Farid Al Tamimi, Sara Abu Aram, Shukran Hmedat, Maimana Abueisha.

**Investigation:** Mohammad Farid Al Tamimi, Sara Abu Aram, Shukran Hmedat, Maimana Abueisha.

**Methodology:** Mohammad Maraqah, Mohammad Farid Al Tamimi, Shukran Hmedat.

**Resources:** Mohammad Maraqah.

**Supervision:** Mohammad Farid Al Tamimi.

**Validation:** Mohammad Maraqah, Mohammad Farid Al Tamimi.

**Writing – original draft:** Mohammad Maraqah, Sara Abu Aram, Shukran Hmedat, Maimana Abueisha.

**Writing – review & editing:** Mohammad Maraqah, Yazan Hmeedat.
